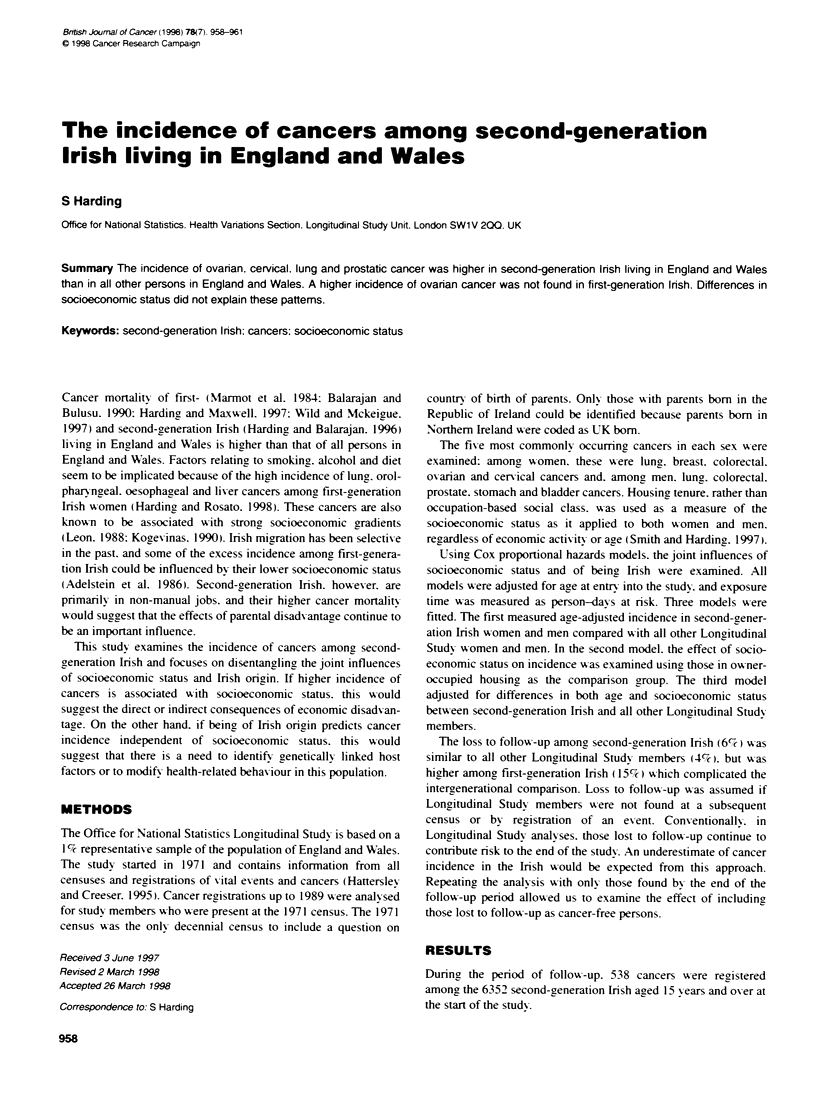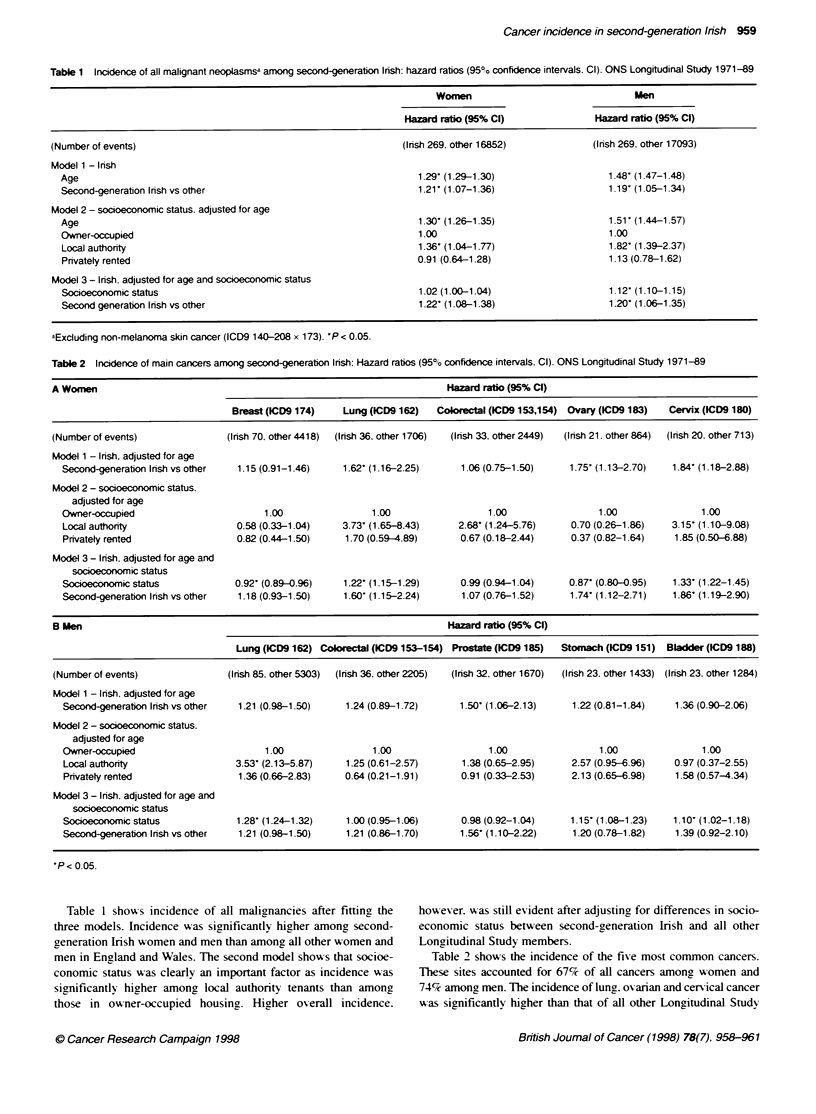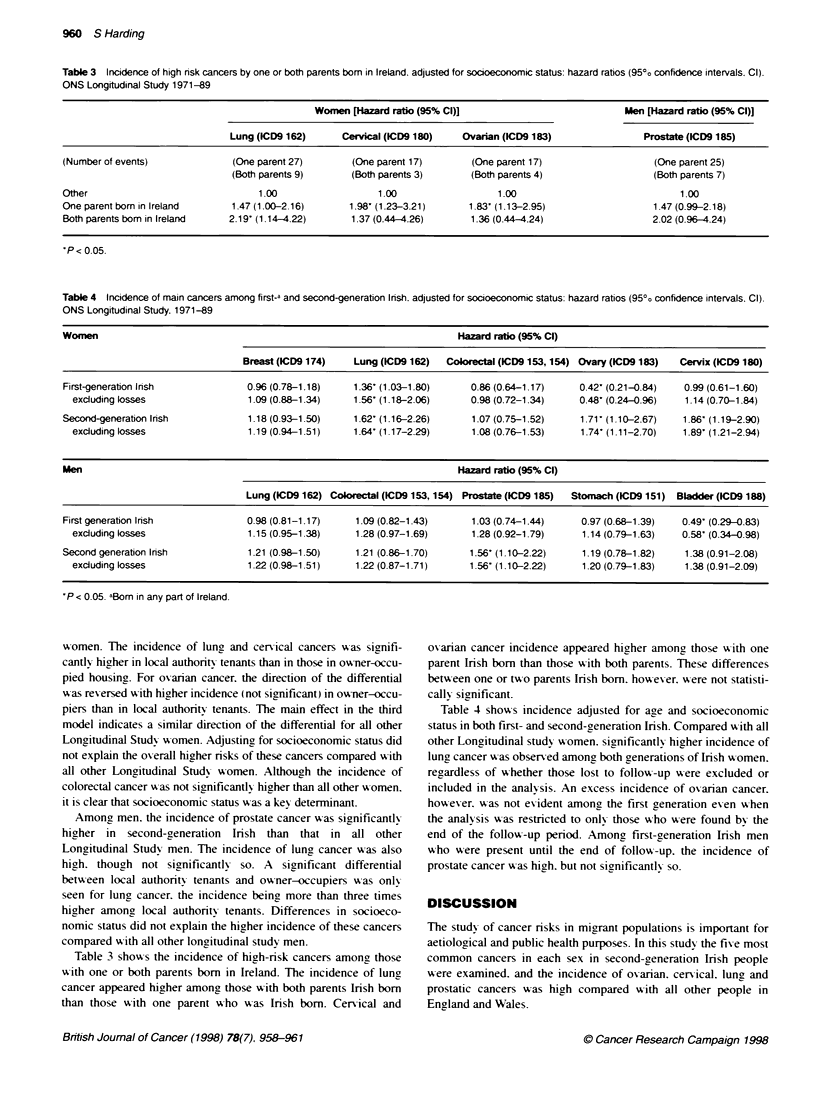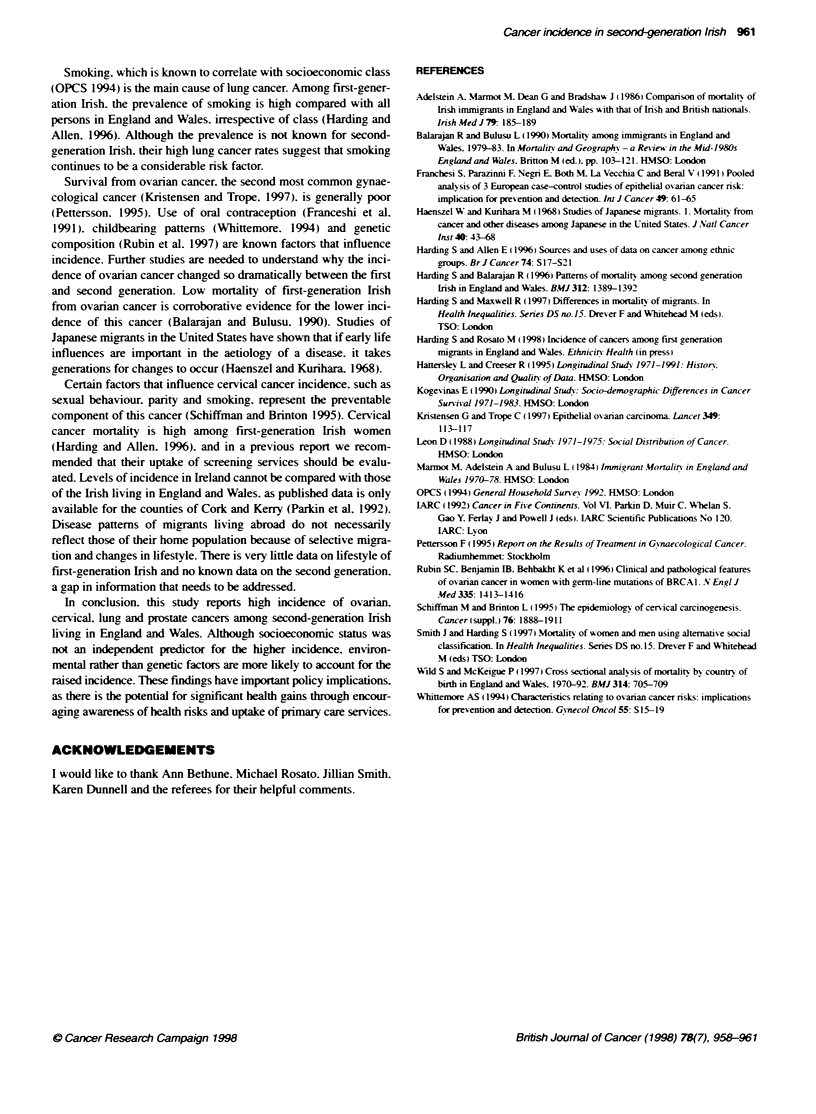# The incidence of cancers among second-generation Irish living in England and Wales.

**DOI:** 10.1038/bjc.1998.608

**Published:** 1998-10

**Authors:** S. Harding

**Affiliations:** Office for National Statistics, Longitudinal Study Unit, London, UK.

## Abstract

The incidence of ovarian, cervical, lung and prostatic cancer was higher in second-generation Irish living in England and Wales than in all other persons in England and Wales. A higher incidence of ovarian cancer was not found in first-generation Irish. Differences in socioeconomic status did not explain these patterns.


British Journal of Cancer (1998) 78(7). 958–961
© 1998 Cancer Research Campaign

### The incidence of cancers among second-generation Irish living in England and Wales

**S Harding**

Office for National Statistics. Health Variations Section. Longitudinal Study Unit. London SW1V 2QQ. UK

**Summary** The incidence of ovarian. cervical. lung and prostatic cancer was higher in second-generation Irish living in England and Wales than in all other persons in England and Wales. A higher incidence of ovarian cancer was not found in first-generation Irish. Differences in socioeconomic status did not explain these patterns.

**Keywords:** second-generation Irish: cancers: socioeconomic status

Cancer mortality of first- (Marmot et al. 1984: Balarajan and Bulusu. 1990: Harding and Maxwell. 1997: Wild and Mckeigue. 1997) and second-generation Irish (Harding and Balarajan. 1996) living in England and Wales is higher than that of all persons in England and Wales. Factors relating to smoking. alcohol and diet seem to be implicated because of the high incidence of lung. orolpharyngeal. oesophageal and liver cancers among first-generation Irish women (Harding and Rosato. 1998). These cancers are also known to be associated with strong socioeconomic gradients (Leon. 1988: Kogevinas. 1990). Irish migration has been selective in the past. and some of the excess incidence among first-generation Irish could be influenced by their lower socioeconomic status (Adelstein et al. 1986). Second-generation Irish. however. are primarily in non-manual jobs. and their higher cancer mortality would suggest that the effects of parental disadvantage continue to be an important influence.

This study examines the incidence of cancers among second-generation Irish and focuses on disentangling the joint influences of socioeconomic status and Irish origin. If higher incidence of cancers is associated with socioeconomic status. this would suggest the direct or indirect consequences of economic disadvantage. On the other hand. if being of Irish origin predicts cancer incidence independent of socioeconomic status. this would suggest that there is a need to identify genetically linked host factors or to modify health-related behaviour in this population.

### METHODS

The Office for National Statistics Longitudinal Study is based on a 1% representative sample of the population of England and Wales. The study started in 1971 and contains information from all censuses and registrations of vital events and cancers (Hattersley and Creeser. 1995). Cancer registrations up to 1989 were analysed for study members who were present at the 1971 census. The 1971 census was the only decennial census to include a question on country of birth of parents. Only those with parents born in the Republic of Ireland could be identified because parents born in Northern Ireland were coded as UK born.

The five most commonly occurring cancers in each sex were examined: among women. these were lung. breast. colorectal. ovarian and cervical cancers and. among men. lung. colorectal. prostate. stomach and bladder cancers. Housing tenure. rather than occupation-based social class. was used as a measure of the socioeconomic status as it applied to both women and men. regardless of economic activity or age (Smith and Harding. 1997).

Using Cox proportional hazards models. the joint influences of socioeconomic status and of being Irish were examined. All models were adjusted for age at entry into the study. and exposure time was measured as person–days at risk. Three models were fitted. The first measured age-adjusted incidence in second-generation Irish women and men compared with all other Longitudinal Study women and men. In the second model. the effect of socioeconomic status on incidence was examined using those in owner-occupied housing as the comparison group. The third model adjusted for differences in both age and socioeconomic status between second-generation Irish and all other Longitudinal Study members.

The loss to follow-up among second-generation Irish (6%) was similar to all other Longitudinal Study members (4%). but was higher among first-generation Irish (15%) which complicated the intergenerational comparison. Loss to follow-up was assumed if Longitudinal Study members were not found at a subsequent census or by registration of an event. Conventionally. in Longitudinal Study analyses. those lost to follow-up continue to contribute risk to the end of the study. An underestimate of cancer incidence in the Irish would be expected from this approach. Repeating the analysis with only those found by the end of the follow-up period allowed us to examine the effect of including those lost to follow-up as cancer-free persons.

### RESULTS

During the period of follow-up. 538 cancers were registered among the 6352 second-generation Irish aged 15 years and over at the start of the study.

*Received 3 June 1997*
*Revised 2 March 1998*
*Accepted 26 March 1998*

*Correspondence to:* S Harding

**Table 1** Incidence of all malignant neoplasms[a] among second-generation Irish: hazard ratios (95% confidence intervals. CI). ONS Longitudinal Study 1971–89

| | Women | Men |
|---|---|---|
| | Hazard ratio (95% CI) | Hazard ratio (95% CI) |
| (Number of events) | (Irish 269. other 16852) | (Irish 269. other 17093) |
| Model 1 – Irish | | |
| Age | 1.29* (1.29–1.30) | 1.48* (1.47–1.48) |
| Second-generation Irish vs other | 1.21* (1.07–1.36) | 1.19* (1.05–1.34) |
| Model 2 – socioeconomic status. adjusted for age | | |
| Age | 1.30* (1.26–1.35) | 1.51* (1.44–1.57) |
| Owner-occupied | 1.00 | 1.00 |
| Local authority | 1.36* (1.04–1.77) | 1.82* (1.39–2.37) |
| Privately rented | 0.91 (0.64–1.28) | 1.13 (0.78–1.62) |
| Model 3 – Irish. adjusted for age and socioeconomic status | | |
| Socioeconomic status | 1.02 (1.00–1.04) | 1.12* (1.10–1.15) |
| Second generation Irish vs other | 1.22* (1.08–1.38) | 1.20* (1.06–1.35) |

[a]Excluding non-melanoma skin cancer (ICD9 140–208 × 173). *$P < 0.05$.

**Table 2** Incidence of main cancers among second-generation Irish: Hazard ratios (95% confidence intervals. CI). ONS Longitudinal Study 1971–89

| A Women | Hazard ratio (95% CI) | | | | |
|---|---|---|---|---|---|
| | Breast (ICD9 174) | Lung (ICD9 162) | Colorectal (ICD9 153,154) | Ovary (ICD9 183) | Cervix (ICD9 180) |
| (Number of events) | (Irish 70. other 4418) | (Irish 36. other 1706) | (Irish 33. other 2449) | (Irish 21. other 864) | (Irish 20. other 713) |
| Model 1 – Irish. adjusted for age | | | | | |
| Second-generation Irish vs other | 1.15 (0.91–1.46) | 1.62* (1.16–2.25) | 1.06 (0.75–1.50) | 1.75* (1.13–2.70) | 1.84* (1.18–2.88) |
| Model 2 – socioeconomic status. adjusted for age | | | | | |
| Owner-occupied | 1.00 | 1.00 | 1.00 | 1.00 | 1.00 |
| Local authority | 0.58 (0.33–1.04) | 3.73* (1.65–8.43) | 2.68* (1.24–5.76) | 0.70 (0.26–1.86) | 3.15* (1.10–9.08) |
| Privately rented | 0.82 (0.44–1.50) | 1.70 (0.59–4.89) | 0.67 (0.18–2.44) | 0.37 (0.82–1.64) | 1.85 (0.50–6.88) |
| Model 3 – Irish. adjusted for age and socioeconomic status | | | | | |
| Socioeconomic status | 0.92* (0.89–0.96) | 1.22* (1.15–1.29) | 0.99 (0.94–1.04) | 0.87* (0.80–0.95) | 1.33* (1.22–1.45) |
| Second-generation Irish vs other | 1.18 (0.93–1.50) | 1.60* (1.15–2.24) | 1.07 (0.76–1.52) | 1.74* (1.12–2.71) | 1.86* (1.19–2.90) |

| B Men | Hazard ratio (95% CI) | | | | |
|---|---|---|---|---|---|
| | Lung (ICD9 162) | Colorectal (ICD9 153–154) | Prostate (ICD9 185) | Stomach (ICD9 151) | Bladder (ICD9 188) |
| (Number of events) | (Irish 85. other 5303) | (Irish 36. other 2205) | (Irish 32. other 1670) | (Irish 23. other 1433) | (Irish 23. other 1284) |
| Model 1 – Irish. adjusted for age | | | | | |
| Second-generation Irish vs other | 1.21 (0.98–1.50) | 1.24 (0.89–1.72) | 1.50* (1.06–2.13) | 1.22 (0.81–1.84) | 1.36 (0.90–2.06) |
| Model 2 – socioeconomic status. adjusted for age | | | | | |
| Owner-occupied | 1.00 | 1.00 | 1.00 | 1.00 | 1.00 |
| Local authority | 3.53* (2.13–5.87) | 1.25 (0.61–2.57) | 1.38 (0.65–2.95) | 2.57 (0.95–6.96) | 0.97 (0.37–2.55) |
| Privately rented | 1.36 (0.66–2.83) | 0.64 (0.21–1.91) | 0.91 (0.33–2.53) | 2.13 (0.65–6.98) | 1.58 (0.57–4.34) |
| Model 3 – Irish. adjusted for age and socioeconomic status | | | | | |
| Socioeconomic status | 1.28* (1.24–1.32) | 1.00 (0.95–1.06) | 0.98 (0.92–1.04) | 1.15* (1.08–1.23) | 1.10* (1.02–1.18) |
| Second-generation Irish vs other | 1.21 (0.98–1.50) | 1.21 (0.86–1.70) | 1.56* (1.10–2.22) | 1.20 (0.78–1.82) | 1.39 (0.92–2.10) |

*$P < 0.05$.

Table 1 shows incidence of all malignancies after fitting the three models. Incidence was significantly higher among second-generation Irish women and men than among all other women and men in England and Wales. The second model shows that socioeconomic status was clearly an important factor as incidence was significantly higher among local authority tenants than among those in owner-occupied housing. Higher overall incidence, however, was still evident after adjusting for differences in socioeconomic status between second-generation Irish and all other Longitudinal Study members.

Table 2 shows the incidence of the five most common cancers. These sites accounted for 67% of all cancers among women and 74% among men. The incidence of lung, ovarian and cervical cancer was significantly higher than that of all other Longitudinal Study

**Table 3** Incidence of high risk cancers by one or both parents born in Ireland. adjusted for socioeconomic status: hazard ratios (95% confidence intervals. CI). ONS Longitudinal Study 1971–89

| | Women [Hazard ratio (95% CI)] | | | Men [Hazard ratio (95% CI)] |
|---|---|---|---|---|
| | Lung (ICD9 162) | Cervical (ICD9 180) | Ovarian (ICD9 183) | Prostate (ICD9 185) |
| (Number of events) | (One parent 27) (Both parents 9) | (One parent 17) (Both parents 3) | (One parent 17) (Both parents 4) | (One parent 25) (Both parents 7) |
| Other | 1.00 | 1.00 | 1.00 | 1.00 |
| One parent born in Ireland | 1.47 (1.00–2.16) | 1.98* (1.23–3.21) | 1.83* (1.13–2.95) | 1.47 (0.99–2.18) |
| Both parents born in Ireland | 2.19* (1.14–4.22) | 1.37 (0.44–4.26) | 1.36 (0.44–4.24) | 2.02 (0.96–4.24) |

*$P < 0.05$.

**Table 4** Incidence of main cancers among first-[a] and second-generation Irish. adjusted for socioeconomic status: hazard ratios (95% confidence intervals. CI). ONS Longitudinal Study. 1971–89

| Women | Hazard ratio (95% CI) | | | | |
|---|---|---|---|---|---|
| | Breast (ICD9 174) | Lung (ICD9 162) | Colorectal (ICD9 153, 154) | Ovary (ICD9 183) | Cervix (ICD9 180) |
| First-generation Irish | 0.96 (0.78–1.18) | 1.36* (1.03–1.80) | 0.86 (0.64–1.17) | 0.42* (0.21–0.84) | 0.99 (0.61–1.60) |
| excluding losses | 1.09 (0.88–1.34) | 1.56* (1.18–2.06) | 0.98 (0.72–1.34) | 0.48* (0.24–0.96) | 1.14 (0.70–1.84) |
| Second-generation Irish | 1.18 (0.93–1.50) | 1.62* (1.16–2.26) | 1.07 (0.75–1.52) | 1.71* (1.10–2.67) | 1.86* (1.19–2.90) |
| excluding losses | 1.19 (0.94–1.51) | 1.64* (1.17–2.29) | 1.08 (0.76–1.53) | 1.74* (1.11–2.70) | 1.89* (1.21–2.94) |

| Men | Hazard ratio (95% CI) | | | | |
|---|---|---|---|---|---|
| | Lung (ICD9 162) | Colorectal (ICD9 153, 154) | Prostate (ICD9 185) | Stomach (ICD9 151) | Bladder (ICD9 188) |
| First generation Irish | 0.98 (0.81–1.17) | 1.09 (0.82–1.43) | 1.03 (0.74–1.44) | 0.97 (0.68–1.39) | 0.49* (0.29–0.83) |
| excluding losses | 1.15 (0.95–1.38) | 1.28 (0.97–1.69) | 1.28 (0.92–1.79) | 1.14 (0.79–1.63) | 0.58* (0.34–0.98) |
| Second generation Irish | 1.21 (0.98–1.50) | 1.21 (0.86–1.70) | 1.56* (1.10–2.22) | 1.19 (0.78–1.82) | 1.38 (0.91–2.08) |
| excluding losses | 1.22 (0.98–1.51) | 1.22 (0.87–1.71) | 1.56* (1.10–2.22) | 1.20 (0.79–1.83) | 1.38 (0.91–2.09) |

*$P < 0.05$. [a]Born in any part of Ireland.

women. The incidence of lung and cervical cancers was significantly higher in local authority tenants than in those in owner-occupied housing. For ovarian cancer, the direction of the differential was reversed with higher incidence (not significant) in owner–occupiers than in local authority tenants. The main effect in the third model indicates a similar direction of the differential for all other Longitudinal Study women. Adjusting for socioeconomic status did not explain the overall higher risks of these cancers compared with all other Longitudinal Study women. Although the incidence of colorectal cancer was not significantly higher than all other women, it is clear that socioeconomic status was a key determinant.

Among men, the incidence of prostate cancer was significantly higher in second-generation Irish than that in all other Longitudinal Study men. The incidence of lung cancer was also high, though not significantly so. A significant differential between local authority tenants and owner–occupiers was only seen for lung cancer, the incidence being more than three times higher among local authority tenants. Differences in socioeconomic status did not explain the higher incidence of these cancers compared with all other longitudinal study men.

Table 3 shows the incidence of high-risk cancers among those with one or both parents born in Ireland. The incidence of lung cancer appeared higher among those with both parents Irish born than those with one parent who was Irish born. Cervical and ovarian cancer incidence appeared higher among those with one parent Irish born than those with both parents. These differences between one or two parents Irish born, however, were not statistically significant.

Table 4 shows incidence adjusted for age and socioeconomic status in both first- and second-generation Irish. Compared with all other Longitudinal study women, significantly higher incidence of lung cancer was observed among both generations of Irish women, regardless of whether those lost to follow-up were excluded or included in the analysis. An excess incidence of ovarian cancer, however, was not evident among the first generation even when the analysis was restricted to only those who were found by the end of the follow-up period. Among first-generation Irish men who were present until the end of follow-up, the incidence of prostate cancer was high, but not significantly so.

### DISCUSSION

The study of cancer risks in migrant populations is important for aetiological and public health purposes. In this study the five most common cancers in each sex in second-generation Irish people were examined, and the incidence of ovarian, cervical, lung and prostatic cancers was high compared with all other people in England and Wales.

© Cancer Research Campaign 1998

Smoking, which is known to correlate with socioeconomic class (OPCS 1994) is the main cause of lung cancer. Among first-generation Irish, the prevalence of smoking is high compared with all persons in England and Wales, irrespective of class (Harding and Allen, 1996). Although the prevalence is not known for second-generation Irish, their high lung cancer rates suggest that smoking continues to be a considerable risk factor.

Survival from ovarian cancer, the second most common gynaecological cancer (Kristensen and Trope, 1997), is generally poor (Pettersson, 1995). Use of oral contraception (Franceshi et al, 1991), childbearing patterns (Whittemore, 1994) and genetic composition (Rubin et al, 1997) are known factors that influence incidence. Further studies are needed to understand why the incidence of ovarian cancer changed so dramatically between the first and second generation. Low mortality of first-generation Irish from ovarian cancer is corroborative evidence for the lower incidence of this cancer (Balarajan and Bulusu, 1990). Studies of Japanese migrants in the United States have shown that if early life influences are important in the aetiology of a disease, it takes generations for changes to occur (Haenszel and Kurihara, 1968).

Certain factors that influence cervical cancer incidence, such as sexual behaviour, parity and smoking, represent the preventable component of this cancer (Schiffman and Brinton 1995). Cervical cancer mortality is high among first-generation Irish women (Harding and Allen, 1996), and in a previous report we recommended that their uptake of screening services should be evaluated. Levels of incidence in Ireland cannot be compared with those of the Irish living in England and Wales, as published data is only available for the counties of Cork and Kerry (Parkin et al, 1992). Disease patterns of migrants living abroad do not necessarily reflect those of their home population because of selective migration and changes in lifestyle. There is very little data on lifestyle of first-generation Irish and no known data on the second generation, a gap in information that needs to be addressed.

In conclusion, this study reports high incidence of ovarian, cervical, lung and prostate cancers among second-generation Irish living in England and Wales. Although socioeconomic status was not an independent predictor for the higher incidence, environmental rather than genetic factors are more likely to account for the raised incidence. These findings have important policy implications, as there is the potential for significant health gains through encouraging awareness of health risks and uptake of primary care services.

### ACKNOWLEDGEMENTS

I would like to thank Ann Bethune, Michael Rosato, Jillian Smith, Karen Dunnell and the referees for their helpful comments.

### REFERENCES

Adelstein A, Marmot M, Dean G and Bradshaw J (1986) Comparison of mortality of Irish immigrants in England and Wales with that of Irish and British nationals. *Irish Med J* **79**: 185–189

Balarajan R and Bulusu L (1990) Mortality among immigrants in England and Wales, 1979–83. In *Mortality and Geography – a Review in the Mid-1980s England and Wales*. Britton M (ed.), pp. 103–121. HMSO: London

Franchesi S, Parazinni F, Negri E, Both M, La Vecchia C and Beral V (1991) Pooled analysis of 3 European case–control studies of epithelial ovarian cancer risk: implication for prevention and detection. *Int J Cancer* **49**: 61–65

Haenszel W and Kurihara M (1968) Studies of Japanese migrants. 1. Mortality from cancer and other diseases among Japanese in the United States. *J Natl Cancer Inst* **40**: 43–68

Harding S and Allen E (1996) Sources and uses of data on cancer among ethnic groups. *Br J Cancer* **74**: S17–S21

Harding S and Balarajan R (1996) Patterns of mortality among second generation Irish in England and Wales. *BMJ* **312**: 1389–1392

Harding S and Maxwell R (1997) Differences in mortality of migrants. In *Health Inequalities. Series DS no.15*. Drever F and Whitehead M (eds). TSO: London

Harding S and Rosato M (1998) Incidence of cancers among first generation migrants in England and Wales. *Ethnicity Health* (in press)

Hattersley L and Creeser R (1995) *Longitudinal Study 1971–1991: History, Organisation and Quality of Data*. HMSO: London

Kogevinas E (1990) *Longitudinal Study: Socio-demographic Differences in Cancer Survival 1971–1983*. HMSO: London

Kristensen G and Trope C (1997) Epithelial ovarian carcinoma. *Lancet* **349**: 113–117

Leon D (1988) *Longitudinal Study 1971–1975: Social Distribution of Cancer*. HMSO: London

Marmot M, Adelstein A and Bulusu L (1984) *Immigrant Mortality in England and Wales 1970–78*. HMSO: London

OPCS (1994) *General Household Survey 1992*. HMSO: London

IARC (1992) *Cancer in Five Continents*. Vol VI. Parkin D, Muir C, Whelan S, Gao Y, Ferlay J and Powell J (eds). IARC Scientific Publications No 120. IARC: Lyon

Pettersson F (1995) *Report on the Results of Treatment in Gynaecological Cancer*. Radiumhemmet: Stockholm

Rubin SC, Benjamin IB, Behbakht K et al (1996) Clinical and pathological features of ovarian cancer in women with germ-line mutations of BRCA1. *N Engl J Med* **335**: 1413–1416

Schiffman M and Brinton L (1995) The epidemiology of cervical carcinogenesis. *Cancer* (suppl.) **76**: 1888–1911

Smith J and Harding S (1997) Mortality of women and men using alternative social classification. In *Health Inequalities*. Series DS no.15. Drever F and Whitehead M (eds) TSO: London

Wild S and McKeigue P (1997) Cross sectional analysis of mortality by country of birth in England and Wales, 1970–92. *BMJ* **314**: 705–709

Whittemore AS (1994) Characteristics relating to ovarian cancer risks: implications for prevention and detection. *Gynecol Oncol* **55**: S15–19

© Cancer Research Campaign 1998